# Autoimmune Hemolytic Anemia and Pulmonary Embolism: An Association to Consider

**DOI:** 10.1055/s-0040-1721733

**Published:** 2021-01-17

**Authors:** Daria Solari, Lorenzo Alberio, Camillo Ribi, Francesco Grandoni, Gregoire Stalder

**Affiliations:** 1 Division and Central Laboratory of Hematology, Department of Oncology, Lausanne University Hospital (CHUV) and University of Lausanne (UNIL), Lausanne, Switzerland; 2 Division of Immunology and Allergy, Department of Medicine, Lausanne University Hospital (CHUV) and University of Lausanne (UNIL), Lausanne, Switzerland

**Keywords:** anticoagulant prophylaxis, autoimmune hemolytic anemia, complication, venous thromboembolism

## Abstract

Autoimmune hemolytic anemia (AIHA) is increasingly recognized as a strong risk factor for venous thrombosis. However, there are currently no guidelines on thromboembolism prevention and management during AIHA. Here, we describe the case of a patient with AIHA and pulmonary embolism and resume the current knowledge on epidemiology, risk factors, treatment, and pathophysiology of thrombosis during AIHA, as well as new therapeutic perspectives to prevent thrombus formation during AIHA.

## Introduction

Autoimmune hemolytic anemia (AIHA) is a rare disease due to the presence of antibodies directed against antigens on the red blood cells (RBCs) surface. The estimated incidence in adults is 1 to 3 in 100,000 per year with a peak age at 60 to 70 years. 1 In two-thirds of the cases, autoantibodies are polyclonal immunoglobulins (Ig) G and, as they react at body temperature, they are named “warm” agglutinins. Roughly half of warm-reactive AIHA (wAIHA) is primary, while the other half is secondary, namely, associated with lymphoproliferative syndromes, solid tumors, autoimmune diseases, infections, or drugs. 2 Signs and symptoms are largely related to anemia. Management remains empirical and is mainly based on the administration of corticosteroids. 3 Although the association between AIHA and venous thromboembolic events (VTE) is increasingly described in the recent literature, 4 to date, no guideline preconizes the introduction of an antithrombotic prophylaxis nor the screening for VTE in AIHA patients. Moreover, the thrombotic risk in AIHA seems to be underestimated by clinicians. 5

Here, we report the case of a 75-year-old man who developed pulmonary embolism (PE) after being diagnosed with idiopathic wAIHA and discuss the current knowledge about thrombotic complications during AIHA.

## Case Report

A 75-year-old man presented to the emergency room with severe shortness of breath. Symptoms had been increasing during the previous 4 weeks, no triggering factors were clearly identified. Dyspnea progressively worsened to a NYHA-III (New York Heart Association III) stage and was not associated with cough nor fever. He had chest pain only under effort, without palpitations, lower limb edema, or orthopnea. Three days prior to admission, he developed severe generalized weakness, dark-colored urine, and jaundice. His medical history was notable for well-controlled moderate chronic obstructive pulmonary disease (COPD; stage II according GOLD [Global Initiative for Chronic Obstructive Lung Disease] classification) and for chronic ischemic heart disease for which he had undergone double coronary artery bypass graft in 2006.

Vital signs on admission included temperature, 37°C; pulse rate, 85 bpm; blood pressure, 131/75 mm Hg; respiratory rate, 15/min; and oxygen saturation, 97% at ambient air.

**Fig. 1:**
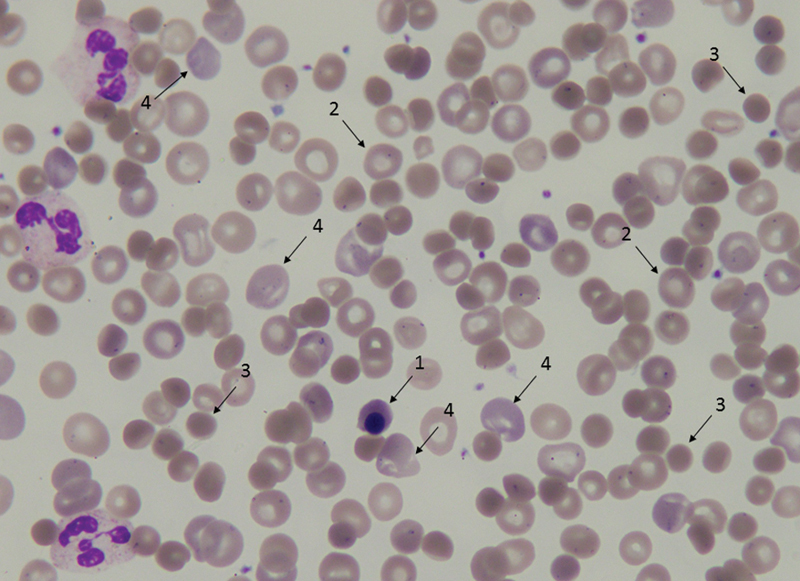
Peripheral blood smear of the patient. Orthochromatic erythroblast (1), normal erythrocytes (2), microspherocytes (3), and polychromatic erythrocytes (4).

Laboratory testing was notable for normochromic, macrocytic anemia with hemoglobin 83 g/L (reference range: 133–177 g/L), absolute reticulocyte count of 614 × 10 ^9^ /L (reference range: 20–120 × 10 ^9^ /L), total bilirubin of 154 μmol/L (reference range: 0–21 μmol/L), direct bilirubin of 13 μmol/L (reference range: 0–10 μmol/L), lactate dehydrogenase of 1,530 U/L (reference range: 135–225 U/L), and undetectable serum haptoglobin (reference range: 0.3–2.0 g/L). Direct antiglobulin test (DAT) was strongly (4 +) reactive for warm IgG1 and IgG3. Dipstick examination did not reveal the presence of hemoglobin in the urine. Peripheral blood smear was characteristic, showing a various degree of anisocytosis, polychromasia, microspherocytes, and few erythroblasts ( Fig. 1 ), and wAIHA was diagnosed.

Workup for underlying causes of wAIHA, including thoracic-abdominal computed tomography (CT) scan, serologic testing for cytomegalovirus (CMV), Epstein–Barr virus (EBV), HIV, viral hepatitis B and C, and screening for monoclonal gammopathy, was negative. Of note, a concomitant autoimmune condition was looked for and only low-titer (1/160) speckled antinuclear antibodies without antibodies to chromatin or extractable nuclear antigens were found. CT scan revealed PE at a right laterobasal segmental and several bilateral subsegmental emboli without evidence of right cardiac overload (no inversion of the right ventricle/left ventricle [RV/LV] ratio). Cardiac enzymes were within normal ranges and echocardiogram showed both normal size and function of LV and RV. No bilateral leg ultrasound was performed. Primary wAIHA with concomitant PE was diagnosed.

Given the history of ischemic heart disease, the patient was transfused with 1 unit packed RBCs. High-dose oral prednisone (1.5 mg/kg per day) was prescribed. Because of ongoing hemolytic anemia, two intravenous steroid pulses (methylprednisolone of 125 mg) were administered additionally on day 9. Given the segmental location of one lesion and many thrombotic risk factors involved (overweight, flare of AIHA, and treatment with high-dose corticosteroids), anticoagulation with rivaroxaban (15-mg twice daily for the first 21 days, afterward 20-mg once a day [od]) was prescribed. The patient was discharged home on day 13 with corticosteroid tapering and rivaroxaban during active hemolysis and for a minimum of 3 months.

## Discussion

### What Is the Risk, Incidence, and Prevalence of Venous Thromboembolism in Autoimmune Hemolytic Anemia?

Among hospitalized patients, those admitted with an immune-mediated disease, including AIHA, are at an increased risk of subsequent VTE. Ramagopalan et al studied the rate ratio of VTE (deep vein thrombosis [DVT] and/or PE) of people admitted to hospital with immune-mediated diseases compared with three references cohorts of hospitalized patients, constructed by identifying the first admission for each individual with various other, mainly minor medical and surgical conditions. 6 They found a rate ratio of respectively 2.83 (95% confidence interval [CI]: 1.62–4.60, *p* < 0.001), 3.64 (2.11–5.85, *p* = 0.001), and 3.83 (3.43–4.25, *p* < 0.001) for patients hospitalized for AIHA compared with controls in each cohort. Zöller et al analyzed the risk for developing PE in patients hospitalized for autoimmune disorders compared with the general Swedish population. They found an incidence ratio (standardized for sex, age, time, and comorbidity) of 11.07 (95% CI: 7.29–16.12) after less than 1 year of follow-up, 3.73 for 1 to 5 years of follow-up (95% CI: 2.58–5.22), 3.16 (95% CI: 1.87–5.00) for 5 to 10 years of follow-up, and an overall incidence ratio of 3.44 (95% CI: 2.74–4.26) for developing PE in patients hospitalized for AIHA. 7

The risk of VTE occurrence among hospitalized adults with selected autoimmune diseases, including AIHA, was examined by Yusuf and colleagues in 2014. 8 Based on almost 6,000,000 observations, they found that, when compared with nonmaternal patients hospitalized without autoimmune disease, patients with AIHA had an adjusted odds ratio for VTE occurring during or before hospitalization of 1.25 (95% CI: 1.05–1.49). 8 Among patients with AIHA, immune thrombocytopenic purpura, rheumatoid arthritis, or systemic lupus erythematosus, or >1 of these diseases, the risk of at least one VTE event was 19.74, 7.72, 4.90, 9.89, and 13.35 per 1,000 person-years, respectively; among the comparison group, the risk was 1.91 per 1,000 person-years. 9

Amid studies of patients with AIHA, 10 11 12 13 14 15 16 17 prevalence of VTE has been reported between 0 and 27% of patients ( Table 1 ).

**Table 1:** Summary of studies on venous thromboembolic events in patients with autoimmune hemolytic anemia

Study	Audia et al 17	Lecouffe-Desprets et al 16	Barcellini et al 15	Roumier et al 14	Baek et al 13	Bongarzoni et al 12	Hendrick 11	Pullarkat et al 10
Year	2018	2015	2014	2014	2011	2005	2003	2002
Design	Retrospective, unicentric, 2006–2016	Retrospective, unicentric, 2009–2013	Retrospective, multicentric, 1978–2013	Retrospective, unicentric, 2001–2011	Retrospective, unicentric, 1994–2010	Prospective, unicentric, 1996–2000	Retrospective, unicentric, 1986–2001	Prospective, multicentric, 1995–2001
Population	wAIHA	wAIHA, excluding cases presenting with solid malignancy, high-grade lymphoproliferative disease, myelodysplatic and/or myeloproliferative disorders	AIHA, only primary	wAIHA	AIHA, excluding patients with drug-induced hemolytic anemia	AIHA, only primary	AIHA with Hb <85 g/L	AIHA
Patients *n* (M/F)	48 (24/24)	40 (13/27)	308 (111/197)	60 (30/30)	32 (1/31)	21 (9/12)	28 (15/13)	30 (11/19)
Primary AIHA/secondary AIHA	23/25	24/16	308/0	23/37	25/7	21/0	7/21	26/4
Follow-up in months a (range)	24.8 (10.1–62.9)	43.2 (1–396)	33 (12–372)	Mean = 46 (6–86)	14 (0.5–238)	Mean = 55 (36–84)	NA	NA
VTE DVT PE DVT and PE Splanchnic thrombosis	11 (23%) b 5 1 5 0	8 (20%) 0 4 4 0	29 (9.4%) 13 11 0 5	12 ^c^ (20%) 1 8 0 4	5 (15.6%) NA NA NA NA	0 (0%) 0 0 0 0	6 (26%) 1 5 0 0	8 (27%) 5 1 2 0
Number of VTE during active hemolysis	10 (91%)	7 (88%)	NA	12 (100%)	NA	–	6 (100%)	7 (88%)
Inpatient/outpatient (at time of VTE)	4/7	2/6	NA	NA	NA	−	NA	NA
Padua’s score	0–5	0–5	NA	NA	NA	NA	NA	NA
Delay between AIHA and VTE diagnosis	Within 2 months for 9, within 1 month for 4. Within the 2 days of hospitalization in eight cases	NA	NA	NA	NA	–	NA	NA
Factors associated with VTE	Icterus, leukocytes, total bilirubin (only bilirubin and leukocytes in multivariate analysis)	Lower hemoglobin level (minimal hemoglobin level)	Thrombotic event (venous and arterial) associated with Hb ≤ 80 g/L and higher LDH level, and postsplenectomy	–	NA	–	NA	Lupus anticoagulant
Factors not associated with VTE	Sex, median age, primary wAIHA, median Padua's score at wAIHA diagnosis, median Padua's score at VTE diagnosis, antiplatelet therapy, VKA, dyspnea, hemoglobin, reticulocytes, elevated LDH, free bilirubin, CRP, CRP at VTE diagnosis, CH50 < N, C3 < N, C4 < N, abnormality of lymphoid lineage on bone marrow aspirate, splenomegaly, hepatomegaly, adenopathy	Age, gender, VTE risk factors, Hb at diagnosis, platelets at diagnosis, primary wAIHA, Evans' syndrome, ≥1 relapse, type of treatment	Anticardiolipin antibodies, lupus anticoagulant	Sex, age, lupus anticoagulant, anticardiolipid antibodies, primary or secondary wAIHA, underlying lymphoma	NA	–	NA	Age, sex, anticardiolipin antibodies, antiphospholipid antibodies
Antithrombotic prophylaxis	2/11 patients with VTE	0/8 patients with VTE “it is routine practice to use thrombotic prophylaxis for inpatients with active AIHA”	NA	NA	NA	NA	1/21 patients with prophylaxis had VTE versus 5/15 patients without prophylaxis ( *p* < 0.1)	NA

Abbreviations: CRP, C-reactive protein; DVT, deep vein thrombosis; F, female; Hb, hemoglobin; LDH, lactate dehydrogenase; M, male; NA, not available; PE, pulmonary embolism; VKA, vitamin-K antagonist; VTE, venous thromboembolism; (w)AIHA, (warm) autoimmune hemolytic anemia.

a Median unless otherwise indicated.

b Postsplenectomy splanchnic thrombosis excluded.

c 12 patients with 13 thrombotic events.

### Can We Predict Which Patient with AIHA Will Have VTE?

Classical thrombotic risk factors, which are utilized in clinical practice to decide when to install prophylactic anticoagulation in medical patients, 18 are frequently absent in AIHA patients with VTE. Two studies found that the majority of VTE in AIHA patients occurred in outpatients with a low Padua's score. 16 17 Interestingly, no constant relationship was found between VTE and antiphospholipid antibodies; either they were absent or they were transient and no thromboembolic event was registered during follow-up. 10 14 15 17 In recent studies with patients with AIHA, between 88 and 100% of VTE episodes occurred during active hemolysis ( Table 1 ). Regarding the lysis parameters, several thresholds, above which the risk of a thrombotic event is higher, have been described. Barcellini et al, on a cohort of 308 patients, observed that VTE was associated with pronounced anemia (hemoglobin level ≤80 g/L, *p* = 0.024) and with a higher median lactate dehydrogenase level (837 U/L vs. 750 U/L, *p* = 0.006). 15 Lecouffe-Desprets et al found that the lowest hemoglobin level during follow-up was lower in patients with VTE (median: 53 vs. 72 g/L, *p* = 0.016). 16 Another study showed that leukocyte count above 7.9 × 10 ^9^ /L (odds ratio [OR] = 15.7, *p* = 0.02) and total bilirubin level of 40 µmol/L or above (OR = 7.4, *p* = 0.02) were predictive for VTE in a multivariate analysis. 17

Since none of these parameters has been validated in a prospective study, caution is needed in their use in clinical practice.

### Usefulness of Anticoagulant Prophylaxis

In 2003, following three cases of fatal PE in patients with AIHA without anticoagulant prophylaxis, Hendrick conducted an audit of the effect of anticoagulant prophylaxis in acute exacerbations of severe autoimmune hemolysis, for which thromboembolic prophylaxis had been introduced on a case-by-case evaluation in 1992. By reviewing a small cohort (23 patients) of cases of AIHA hospitalized between 1986 and 2001, he found that 1 out of 21 patients with prophylaxis had VTE versus 5 out of 15 patients without prophylaxis. 11 In the studies of Audia et al and Lecouffe-Desprets et al, prophylactic anticoagulation was present in 2 out of 11 and 0 out of 8 patients with VTE, respectively. 16 17 Despite their numerous limitations, these studies suggest that prophylaxis is useful and, in absence of randomized clinical trials, should be proposed in both in- and outpatients with a flare of AIHA.

### What Are the Postulated Mechanisms Predisposing to VTE in AIHA?

**Fig. 2:**
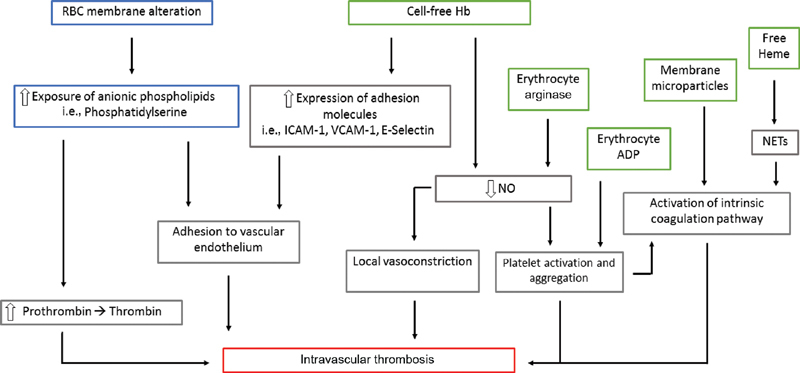
Postulated model of thrombus formation during hemolysis. Modification of RBC membrane (blue), release of intra RBC molecules (green). ADP, adenosine diphosphate; Hb, hemoglobin; ICAM-1, intracellular adhesion molecule 1; NETs, neutrophil extracellular traps; NO, nitric oxide; RBC, red blood cell; VCAM-1, vascular cell adhesion molecule 1.

Multiple mechanisms have been suggested to explain the pathogenesis of the procoagulant condition associated with hemolysis ( Fig. 2 ). RBC membrane is altered by autoantibodies. This leads to an increased exposure of anionic phospholipids, particularly phosphatidylserine (PS). The anionic surface of PS promotes the assembly of enzymatic complexes of coagulation, enhancing the conversion of coagulation factor X to Xa and of prothrombin to thrombin. In sickle-cell disease, it has also been shown that PS-positive RBCs adhere more strongly to vascular endothelium. 19 Destruction of red-cell membrane also leads to the release of microparticles (MPs). MPs that carry negatively charged PS promote thrombin generation. Heme-laden MPs can transfer heme to vascular endothelium and mediate oxidative stress, vascular dysfunction, and occlusion. 20 Destruction of erythrocytes leads also to the release in the circulation of cell-free hemoglobin, erythrocyte arginase, free heme, and ADP. ADP induces platelet shape change, secretion of storage granules, influx and intracellular mobilization of Ca ^2+^ , and inhibition of stimulated adenyl-cyclase activity, thus promoting platelet aggregation. 21 Cell-free hemoglobin enhances the expression of adhesion molecules, such as intracellular adhesion molecule 1 (ICAM-1), vascular cell adhesion molecule 1 (VCAM-1), and E-selectin on endothelial cells. 22 Erythrocyte arginase, together with cell-free hemoglobin, decreases nitric oxide (NO) availability by scavenging and decreasing its synthesis. This results in reduced cyclic guanine monophosphate production (cGMP), leading to impaired regulation of smooth muscle tone and platelets activation. Finally, free heme, has per se proinflammatory effects on endothelial cells but seems also to trigger neutrophil extracellular traps (NETs) formation, which recruit RBCs, activate platelets, and promote fibrin deposition via activation of the contact phase of coagulation. 23 24 Besides the mechanisms described above, treatment itself (in particular glucocorticoids) may contribute to the prothrombotic state in patients with AIHA. A population-based case-control study investigated the risk of VTE related to glucocorticoid use by comparing 38,765 cases of VTE to 387,650 matched controls for age and gender extracted from Danish national registries. 25 The authors found that treatment with systemic glucocorticoids increased the risk of VTE, particularly PE. Glucocorticoid use increased VTE risk if “present” (most recent prescription for glucocorticoids within 90 days before the VTE = adjusted incidence rate ratio [IRR] = 2.31; 95% CI: 2.18–2.45), “new” (first-ever prescription within 90 days before the VTE = 3.06; 2.77–3.38), “continuing” (first-ever prescription period more than 90 days before VTE = 2.02; 1.88–2.17), or “recent” (most recent prescription finished between 91 and 365 days before VTE = 1.18; 1.10–1.26). Furthermore, adjusted incidence rate ratio increased with total prednisone equivalent dose. 25 However, the results of this study were not free of possible residual confounding factors, such as an inflammatory flare, chemotherapies, or cancer. 26 Similarly, studies on the impact of glucocorticoids on procoagulant, anticoagulant, and fibrinolytic factors showed differential biochemical effects depending on the clinical situation. 27 For example, in healthy patients, glucocorticoids increase factors VII, VIII, and XI activity, while during active inflammation, glucocorticoids increased the levels of plasminogen activator-inhibitor 1 and decreased von Willebrand factor and fibrinogen levels, thus theoretically decreasing the prothrombotic state induced by inflammation itself. Of note, these were not clinical outcome studies.

### Perspectives

Based on the pathophysiology of thrombus formation during AIHA, more targeted therapies to minimize the risk of bleeding are currently explored. Erythrocyte-derived ADP induces significant platelet activation that could be prevented by an ADP P2Y _12_ receptor inhibitor. This was demonstrated, with cangrelor, in an in vitro study ran by Gremmel et al. 28 Because of its selective importance in thrombus formation while being dispensable for hemostasis, FXII is another interesting target. 29 Moreover, FXIIa triggers inflammation through the activation of the bradykinin-producing kallikrein-kinin system. Its inhibition could have an additional anti-inflammatory effect that could be beneficial in hemolytic anemia associated with autoimmune conditions. 29

## Conclusion

To date, no guideline preconizes the introduction of an antithrombotic prophylaxis nor the screening of VTE in patients suffering of AIHA.

However, based on the scarce evidence in the literature and the postulated pathophysiology of thrombosis in hemolytic anemias, it seems reasonable to suggest a high level of suspicion for VTE in patients with AIHA flare and to prescribe anticoagulant prophylaxis while the patient is exhibiting frank hemolysis. Numerous questions are still unanswered. Should we screen every patient with AIHA for VTE? Could D-dimer levels help in this decision? Are there indicators for the need of thrombotic prophylaxis in AIHA? Which type of antithrombotic agent will suits best for prophylaxis and treatment, respectively? Which duration of anticoagulation is needed? Due to the rarity of AIHA, the responses will need prospective multicentric studies and time. For the time being, clinicians have to be aware of the increased thrombotic risk of AIHA patients and individualize management decisions.
